# Hypertrophic, Dilated, and Arrhythmogenic Cardiomyopathy: Where Are We?

**DOI:** 10.3390/biomedicines11020524

**Published:** 2023-02-11

**Authors:** Hamza El Hadi, Anne Freund, Steffen Desch, Holger Thiele, Nicolas Majunke

**Affiliations:** Department of Internal Medicine/Cardiology, Heart Center Leipzig, University of Leipzig, Strümpellstr. 39, 04289 Leipzig, Germany

**Keywords:** hypertrophic cardiomyopathy, dilated cardiomyopathy, arrhythmogenic cardiomyopathy, heart failure, sudden cardiac death

## Abstract

Cardiomyopathies are a heterogeneous group of structural, mechanical, and electrical heart muscle disorders which often correlate with life-threatening arrhythmias and progressive heart failure accounting for significant cardiovascular morbidity and mortality. Currently, cardiomyopathies still represent a leading reason for heart transplantation worldwide. The last years have brought remarkable advances in the field of cardiomyopathies especially in terms of understanding the molecular basis as well as the diagnostic evaluation and management. Although most cardiomyopathy treatments had long focused on symptom management, much of the current research efforts aim to identify and act on the disease-driving mechanisms. Regarding risk assessment and primary prevention of sudden cardiac death, additional data are still pending in order to pave the way for a more refined and early patient selection for defibrillator implantation. This review summarizes the current knowledge of hypertrophic, dilated and arrhythmogenic cardiomyopathy with a particular emphasis on their pathophysiology, clinical features, and diagnostic approach. Furthermore, the relevant ongoing studies investigating novel management approaches and main gaps in knowledge are highlighted.

## 1. Introduction

Cardiomyopathies encompass a diverse group of diseases characterized by structural and functional disorders of the heart in the absence of coronary artery disease, hypertension, valvular disease, and congenital heart disease sufficient to cause the observed myocardial abnormality. They can be either inherited and/or acquired and represent a leading cause of heart failure-related morbidity and mortality. Cardiomyopathies can present with heterogeneous clinical courses, ranging from an asymptomatic to a severely symptomatic phenotype which can present with heart failure, malignant arrhythmias, or sudden cardiac death [1]. During the past few years, there have been unprecedented improvements regarding the clinical phenotyping, imaging approaches, and genetics of cardiomyopathies. This has contributed to a better understanding of disease pathogenesis and consequently allowed the implementation of optimal therapeutic strategies. In the present review, we focus on three cardiomyopathies: dilated cardiomyopathy (DCM), hypertrophic cardiomyopathy (HCM), and arrhythmogenic cardiomyopathy (ACM). Herein we aim to provide a concise but practical overview of their pathogenesis, clinical features, diagnostic work-up, management, and key aspects worthy of further investigation. Restrictive cardiomyopathy presenting mainly as a secondary manifestation of systemic diseases was not included within the scope of the present review paper.

## 2. Hypertrophic Cardiomyopathy

HCM is the most common inherited cardiomyopathy. It involves mutations in numerous genes encoding sarcomeric proteins and is transmitted in an autosomal dominant manner with variable penetrance. Historically, HCM was first defined in 1868 by Vulpian et al. as an idiopathic hypertrophic subaortic stenosis [2]. In the late 1950s, several reports emerged describing asymmetrical hypertrophy or muscular hamartoma of the heart in young people with sudden death [3]. Few years later Morrow and Braunwald described using invasive hemodynamics high subaortic gradients in patients with left ventricular (LV) hypertrophy without having subaortic membranous stenosis [4].

HCM is defined as the presence of an increase in wall thickness (greater than 14 mm) in one or more LV segments, as measured by an imaging technique, which cannot be justified by abnormal loading conditions. The estimated prevalence according to epidemiological studies ranges from 1:200 to 1:500 with an annual mortality rate of approximately 1.4% [5].

### 2.1. Etiology and Pathophysiology

Despite the fact that in about 40% of HCM patients the causal genes remain unidentified, it is considered a familial disease mainly resulting from autosomal dominant mutations of genes encoding proteins of myofilament contractile components of the cardiac sarcomere or Z-disc and calcium-controlling proteins (Table 1). Although over 2000 different mutations have been described, pathogenic variants in the beta-myosin heavy chain (MYH7) gene and myosin binding protein C (MYBPC3) gene account for the most disease-causing mutations [6]. The consequent genetic mutations induce an energetic imbalance in cardiomyocytes and impairment of calcium homeostasis which lead in turn to several downstream effects including myofibril disruption, hypertrophy, and fibrosis formation [7].

The switch of energy substrates has been suggested to play a central role in the structural cardiac remodeling in HCM. Early reports had supported a relationship between inefficient myocardial energetics and a ‘compensatory’ cardiac hypertrophy and diastolic dysfunction in HCM [8]. Recent findings confirmed a relevant reduction of myocardial efficiency mainly in symptomatic HCM, which at least in part resulted from a metabolic shift towards glucose oxidation [9]. Additional data from nuclear cardiac imaging supported the presence of an extensive shift towards glucose oxidation in the myocardium of symptomatic HCM patients with secondary increase of septal mass and LV outflow gradient [10].

Moreover, in HCM animal models the expression of fatty acids transporter CD36 was reduced, suggesting an impaired myocardial fatty acids uptake. Changes in CD36 availability were also observed in humans with hereditary HCM, confirming the cardiac metabolic shift from fatty acids towards glucose utilization [11].

Myocardial ischemia is another characteristic feature in HCM. Several mechanisms have been described such as an increase in muscle mass, presence of coronary microvascular dysfunction, impaired LV filling due to increased ventricular diastolic pressures, and decreased arteriolar lumen due to thickening of the coronary artery walls [12]. The morphologic pattern of hypertrophy in HCM is variable and can affect any LV segment. The asymmetric septal hypertrophy represents the most common appearance. However, other phenotypic patterns including diffuse concentric, apical, and midventricular hypertrophy were reported [13].

The occurrence of obstruction within the LV outflow tract (LVOT) represents a hallmark in the pathophysiology of HCM and has been shown to be independently associated with adverse HCM-related outcomes. The hypertrophy of the basal septum favors a systolic traction (Venturi effect) of the anterior mitral valve leaflet (SAM) towards the hypertrophied interventricular septum, resulting in subaortic systolic gradient and narrowing of LVOT. The geometry of the outflow tract, degree of basal septal hypertrophy, abnormalities in mitral valve architecture and its subvalvular apparatus, as well as the loading condition (preload and afterload) play an important role in the presence or absence of LVOT obstruction [14]. The presence of intracavitary systolic obstruction is estimated to be present at rest or with provocative maneuvers in approximately 70% of HCM patients [15].

### 2.2. Clinical Manifestation

HCM may manifest at any phase of life from the infant to elderly age, but most commonly occurs in the third decade of life. The majority of patients with nonobstructive disease may remain asymptomatic or develop only minor symptoms, maintaining a normal life expectancy and a benign clinical course. HCM symptoms, which include exertional dyspnea and chest pain, are mainly related to heart failure. Less commonly, HCM patients report presyncope/syncope episodes and palpitations due to supraventricular or ventricular arrhythmias. A minority of patients may develop progressive heart failure, which is usually associated with poor prognosis [16]. Sudden cardiac death (SCD) is the most appalling complication in HCM which usually affects asymptomatic or oligosymptomatic individuals under 30 years old and is primarily mediated by malignant ventricular arrhythmias, which can be a consequence of myocardial ischemia, LVOT obstruction, or supraventricular arrhythmias [17].

### 2.3. Diagnosis

#### 2.3.1. Electrocardiogram and Dynamic Holter

The electrocardiogram, despite its low specificity, demonstrates abnormalities in the majority of HCM patients. The most common electrocardiographic findings include LV hypertrophy by voltage criteria, left axis deviation, P wave abnormalities (due to left and/or right atrial enlargement), prominent Q-waves in inferolateral leads, and presence of repolarization anomalies (T-wave inversions) [18]. Furthermore, 24–48-h Holter is essential to detect ventricular arrhythmias and subsequently risk stratification of SCD [19].

#### 2.3.2. Echocardiography

Transthoracic echocardiography is the first-line imaging approach in the diagnosis of HCM through the assessment of LV wall thickness. Right ventricular hypertrophy is a common feature in more than 50% of HCM patients, and is usually associated with a worse prognosis [20]. In the majority of cases, the LV ejection fraction (LVEF) is normal or supranormal due to complex LV structure modifications. For these reasons, the assessment of myocardial deformation through quantification of strain using tissue Doppler imaging (TDI) or 2D-derived speckle tracking echocardiography can better detect early systolic ventricular abnormalities and predict clinical outcomes in patients with HCM [21,22]. In addition, the echocardiographic examination at rest or during provocative maneuvers (standing/exercise/Valsalva) provides indispensable information regarding the presence of SAM as well as other anomalies of the mitral valve including chordal elongation, excessive leaflet tissue, prolapse and direct insertion of the papillary muscle into the anterior leaflet. In this context, LVOT obstruction is often defined on the basis of echocardiography when the pressure gradient across the LVOT is at least 30 mmHg at rest or during physiological provocation. Furthermore, diastolic dysfunction is usually an early echocardiographic finding and one of the main drivers involved in heart failure in patients with HCM. This include an abnormal transmitral flow, increase in left atrium indexed volume, and a decrease in early diastolic myocardial velocity (e’) [23].

#### 2.3.3. Cardiac Magnetic Resonance Imaging

According to the current guidelines of the American College of Cardiology (ACC), American Heart Association (AHA), and European Society of Cardiology (ESC), cardiac magnetic resonance (CMR) with late gadolinium enhancement (LGE) is recommended (Class I-recommendation) for the diagnostic work-up of HCM [24,25]. Compared to echocardiography, it allows for the determination the LV wall thickness with higher accuracy and the identification of apical aneurysms, which have been shown to be associated with greater risk of SCD [26]. Furthermore, CMR using gadolinium contrast is considered the reference standard in the non-invasive assessment of myocardial fibrosis, which can be detected in about 75% of patients with HCM [27]. The presence and extension of myocardial fibrosis detected by LGE on CMR has been shown to be an independent predictor of adverse cardiac outcomes such as SCD, arrhythmia, and heart failure in HCM patients [28]. In the context of prognostic utility, the extension rather than the presence of LGE has recently been shown to have an incremental value in terms of SCD risk stratification. In particular, LGE extension greater than 15% of the myocardial mass in patients with HCM is associated with a 3-fold increased risk of SCD [29].

#### 2.3.4. Genetic Counselling and Testing

The main aim of genetic testing is to allow the identification of asymptomatic individuals and thus implementation of primary prevention approaches in high-risk individuals. Current clinical guidelines recommend genetic testing and counselling in all patients with clinical diagnosis of HCM and their first-degree adult relatives (Class I-recommendation) [24,25]. Despite the fact that a positive genetic test constitutes a small proportion of the HCM population due to the variable penetrance of mutations underlying HCM, it can firmly support the diagnosis in unclear or atypical clinical presentations of HCM. First-degree genotype-positive relatives can continue ongoing clinical surveillance [30]. Another implication of genetic testing lies in the differential diagnosis with other disorders associated with LV hypertrophy that can mimic HCM and require different management (Fabry disease, Noonan Syndrome, glycogen storage cardiomyopathy) [31].

### 2.4. Management Aspects

#### 2.4.1. Lifestyle Modification

Considering the marked benefits of regular exercise and avoidance of a sedentary lifestyle, determining safe levels of athletic participation is still challenging. Recent data suggest that even vigorous exercise in low-risk athletes with HCM is not associated with ventricular arrhythmias. Therefore, participation in high-intensity exercise may be considered in asymptomatic HCM patients with low-risk profile (Class IIb-recommendation) [24]. In addition, triggers causing a reduction in cardiac preload and an exacerbation of obstruction such as dehydrating situations and excessive alcohol intake should be avoided.

#### 2.4.2. Pharmacologic Therapy

**Figure 1:**
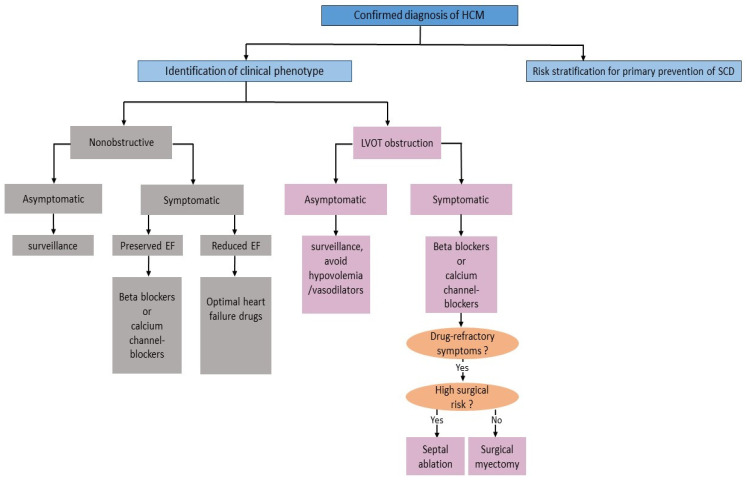
Problem-oriented management of HCM based on clinical phenotype and stage of disease. HCM: hypertrophic cardiomyopathy; SCD: sudden cardiac death; EF: ejection of fraction; LVOT: left ventricular outflow tract.

The conventional pharmacological approaches in HCM aim to improve symptoms and quality of life. Non-vasodilating beta-blockers (as metoprolol, atenolol, and propranolol) are considered the first-line therapy in symptomatic HCM. Their efficacy is a result of negative chronotropy and inotropy, reduced LVOT gradients, increased systolic ejection time, and improved diastolic filling. If beta-blockers are contraindicated, ineffective or not well tolerated, non-dihydropyridine calcium channel blockers such as verapamil and diltiazem can improve symptoms by inducing a negative inotropic effect and prolonging the LV filling time. In case of exertional dyspnea, low-dose loop or thiazide diuretics might be considered but should be used cautiously since they could promote obstruction by decreasing preload (Figure 1) [32]. Disopyramide, which was historically introduced as an antiarrhythmic agent, has been shown efficacy in the treatment of HCM because of its potent negative inotropic properties resulting in reducing the LVOT gradients and relief of symptoms. Accordingly, current guidelines of the ESC and AHA/ACC assigned to disopyramide a class I and IIa recommendation, respectively, in combination with a beta-blocker or verapamil in case of symptom persistence [25,33].

#### 2.4.3. Septal Reduction Therapy

Septal reduction therapy at experienced centers is indicated in patients with a resting or provoked obstructive disease who remain symptomatic despite optimal medical therapy (Class I-recommendation) (Figure 1) [33]. Compared to surgical myectomy (Morrow procedure), transcatheter ablation of the septum with ethanol has demonstrated in the last three decades an increasing efficacy and safety, proposing it as a valid alternative to surgical septal myectomy. However, to date there have been no completed randomized clinical trials comparing the clinical outcomes of surgical myectomy and alcohol septal ablation and consequently the current recommendations are based on retrospective data and meta-analyses of observational studies [33,34,35]. The latest ESC guidelines gave no definitive preference in the choice of the septal reduction therapeutic approach, but encouraged taking consideration the cardiac features, concomitant morbidities, and preference as well as the center experience [33]. In this regard, surgical septal myectomy rather than transcatheter septal ablation was assigned class I recommendation in presence of other lesions requiring surgical intervention (e.g., mitral valve or papillary muscle repair), or a pre-existing left bundle branch block [33].

#### 2.4.4. Risk Stratification and Primary Prevention of SCD

According to the latest European guidelines, a 5-year-SCD assessment is recommended (Class I-recommendation) in all HCM patients in order to identify candidates who will benefit from primary prevention using an implantable cardioverter-defibrillator (ICD) [24]. To this aim a risk stratification algorithm taking into account age, family history of SCD, LV wall thickness, left atrial diameter, LVOT gradient, documented non-sustained ventricular tachycardia, and personal history of unexplained syncope has been developed. Accordingly, ICD implantation should be considered in patients aged 16 years or more with an estimated 5-year risk score of at least 6% (Class IIa recommendation). In patients with intermediate or low estimated SCD-risk, primary SCD-prevention should be considered only after taking into consideration additional factors as presence of significant LGE or LV apical aneurysm at CMR, reduced LVEF, presence of sarcomeric mutations, and blood pressure response during exercise test [24]. Recently, an SCD risk prediction model in pediatric (HCM Risk-Kids model) was validated [36]. This model provides a support method to facilitate SCD risk prediction and improve ICD use in high-risk pediatric patients.

### 2.5. Emerging Therapeutic Approaches for HCM

Advances in understanding the genetic mechanisms of HCM has led to an increasing interest to develop targeted molecular therapies which improve symptoms and positively influence the disease course (Table 2).

#### 2.5.1. Modulators of Cardiac Myosin

Recently, mavacamten (MYK-461) has emerged as a novel allosteric inhibitor of cardiac myosin adenosine) triphosphatase (ATPase) sarcomere level, causing a reduction of the cardiomyocyte hypercontractility and promoting cardiac muscle relaxation. Its beneficial effects on HCM patients were first shown in the phase 2 PIONEER-HCM trial. The results were promising since mavacamten was associated with improvement of post-exercise LVOT-gradient and exercise capacity in patients with obstructive HCM [37]. Following this positive outcome, mavacamten was further studies in a multicenter, phase 3, randomized, double-blind, placebo-controlled trial in 251 patients with severe symptomatic obstructive HCM. Patients were randomized to mavacamten or placebo on top of their previous beta-blocker or calcium channel blocker therapy. After 30 weeks of treatment, the primary endpoint was reached by conferring a significant improvement of functional capacity assessed by cardiopulmonary exercise test and heart failure symptoms according to the New York Heart Association (NYHA) classification in the mavacamten arm [38]. In nonobstructive HCM, mavacamten through the MAVERICK trial has been demonstrated to be well tolerated and associated with improvement of heart failure markers (NT-proBNP and Troponin) [39]. Furthermore, recent findings deriving from the phase 3 clinical trial VALOR-HCM demonstrated that treatment with mavacamten for 16 weeks improved the symptoms and reduced eligibility for needing septal reduction therapy among symptomatic patients with obstructive HCM [40]. Based on these findings, in April 2022, mavacamten was approved by the U.S. Food and Drug administration (FDA) for the treatment of adult patients with symptomatic NYHA class II-III obstructive HCM. Aficamten is a next-generation cardiac myosin inhibitor with similar mechanism of action to mavacamten, but has a shorter half-life and a wider therapeutic response [41]. A randomized phase 2 trial of aficamten (REDWOOD-HCM), demonstrated that treatment with aficamten for 10 weeks resulted in significant reductions in the LVOT gradients compared to placebo [42]. In addition, promising results are still pending from the ongoing phase 3 randomized, placebo-controlled, international clinical trial (SEQUOIA-HCM) designed to evaluate aficamten in symptomatic obstructive HCM despite medical therapy with disopyramide [43].

#### 2.5.2. Ion Channel Inhibitors

HCM cardiomyocytes are characterized by an enhanced late sodium current activity, leading to increased levels of intracellular calcium which ultimately contribute to arrhythmic propensity. Moreover, the intracellular sodium overload promotes the cardiomyocyte diastolic dysfunction and impaired myocardial perfusion [44,45]. Ranolazine is an inhibitor of late sodium current which has been shown to improve the diastolic function and reduce the arrhythmogenicity of HCM cardiomyocytes. The beneficial cardiovascular effect of this drug was also reported in other tissues as pancreatic islets inducing a lowering of fasting plasma glucose and hemoglobin A1c (HbA1c) levels [46]. Based on this rationale, a double-blind, placebo-controlled randomized trial (RESTYLE-HCM) enrolled 80 nonobstructive HCM patients which were randomized to placebo or ranolazine. Despite the fact that ranolazine induced no significant benefits in terms of functional capacity (peak oxygen consumption during exercise), notably this drug was associated with relevant reduction of arrhythmic burden compared to placebo [47]. Similarly, the LIBERTY-HCM trial failed to demonstrate an improvement of symptoms and exercise capacity in patients with HCM following a treatment with the more potent late sodium current activity inhibitor eleclazine [48]. All together, despite the limited clinical relevance of late sodium current blockers in improving the symptoms and exercise capacity of HCM patients, the findings of the RESTYLE-HCM trial highlighted the potential of ranolazine as a possible future antiarrhythmic agent to reduce the occurrence of arrhythmias in HCM.

#### 2.5.3. Myocardial Metabolism Modulators

The inefficient phosphate metabolism observed in HCM patients is another target of novel therapies. Perhexiline (a Carnitine Palmitoyltransferase-1 Inhibitor) was initially utilized as an antianginal treatment because of its coronary dilatator effects. Successively, this agent had demonstrated positive effects on the myocardial energetic status by shifting the myocardial metabolism from fatty acid to glucose utilization and consequently ameliorating the myocardial efficiency by increasing ATP production [49]. This observation was the corner stone of the METAL-HCM trial where perhexiline, compared to placebo, demonstrated an improvement of cardiac energetic impairment, diastolic function, and exercise performance in obstructive HCM [50]. Despite these preliminary findings, the phase-2 open-label study in HCM patients with moderate to advanced heart failure was discontinued early due to lack of therapeutic efficacy. More recently, the IMPROVE-HCM trial was designed to investigate the safety and efficacy of a novel mitotropic agent IMB-101 in patients with nonobstructive HCM [51]. IMB-101 has been shown to be able to increase the myocardial efficiency by reducing fatty acid oxidation and promoting glucose utilization [52].

## 3. Dilated Cardiomyopathy

DCM is defined as dilatation of the left or both ventricles and systolic dysfunction in the absence of abnormal loading conditions (as hypertensive, valvular, congenital heart disease) or significant coronary artery disease sufficient to explain a global systolic impairment [53]. DCM represents a leading cause of heart transplantation (HTx) due to heart failure with an estimated prevalence of 1:250 to 1:500 in the general population. Though DCM can occur at any age, it manifests more frequently in the third or fourth decade of life [54].

### 3.1. Etiology and Pathophysiology

The causes of DCM are heterogeneous and can be broadly classified into genetic and nongenetic. Gene mutations occur in up to 40% of DCM cases and usually involve genes encoding for cytoskeletal, sarcomere and nuclear envelope proteins [55]. Interestingly, pathogenic genetic variants are identified in more than 10% of non-familial DCM cases [56]. Nongenetic triggers include pathologies that mediate inflammatory processes (myocarditis, autoimmune diseases), toxic influences (chemotoxines, drugs) and metabolic disorders.

#### 3.1.1. Genetic Causes of DCM

More than 50 DCM-related genes have been shown to be implicated in the pathogenesis of DCM. Most of these genetic alterations have an autosomal dominant inheritance pattern; however, other patterns have been reported including autosomal recessive, x-linked, mitochondrial and polygenic [57]. Mutation of the titin (TTN) gene has been shown to be the leading genetic cause of DCM, and is prevalent in up to 25% of cases. TTN gene is present on chromosome 2 and encodes for the large sarcomere cytoskeleton protein titin, which connects actin and myosin within the myocardial cells [58].

About 5% of DCM cases are linked to mutations in the Lamin A/C (LMNA) gene encoding to lamin proteins A and C involved in the stabilization of the inner nuclear lamina in cardiomyocytes. Notably, LMNA mutations have been shown to be associated with malignant ventricular arrhythmias and a high incidence of SCD. Moreover, LMNA mutations have been described in patients with muscular dystrophy Emery-Dreyfus (EDMD), limb-girdle-dystrophy, axonal neuropathy type 2 Charcot- Marie-Tooth, and familiar partial lipodystrophy [59]. Other mutations commonly associated with DCM have been identified in the genes encoding for Phospholamban (PLN), Filamin C (FLNC), desmin (DES), dystrophin (DMD), actin-alpha cardiac muscle (ACTC1), troponin T (TNNT2), troponin I (TNNI3), cardiac isoforms of MYH7, and delta-sarcoglycan (SGCD) (Table 1) [60].

To date, mutations in metabolic genes as a direct cause of DCM have not yet been reported in humans. However, recent findings from an animal model showed that suppression of cardiac low-density lipoprotein receptor-related protein 6 (LRP6) expression is associated with altered cardiac fatty acid utilization and lethal DCM. Subsequently, further human studies to determine the extent of contribution of fatty acids metabolism in the pathogenesis of DCM could help to elucidate disease pathogenic events [61].

#### 3.1.2. Nongenetic Causes of DCM

##### Myocarditis

Although myocarditis usually heals completely, it is reported that up to 6–30% of cases may shift in DCM with worsening of cardiac function [62]. Myocarditis may evolve into a chronic low-grade inflammatory state mediated by a cascade of proinflammatory cytokines leading to myocyte necrosis, myocardial scarring and left ventricular dilatation [53]. Viral infection is the most common cause of myocarditis in developed countries. Among the cardiotropic viruses the most cited are enteroviruses (mainly coxsackievirus) adenovirus, parvovirus B19, herpesviruses, human cytomegalovirus, Epstein Barr virus, and less commonly hepatitis virus [63]. Besides cardiotropic viruses, myocarditis can be caused by a wide spectrum of pathogens including bacteria, fungi and parasites. In the same context, there is growing evidence that links the novel coronavirus disease 2019 (COVID-19) with myocardial injury which may evolve into DCM [64]. It was suggested that cytokine storm induced by the strong release of inflammatory cytokines and chemokines is strongly involved in the pathogenesis of COVID-19- related cardiomyopathy [65].

##### Drugs and Cardiotoxins

In the western countries, excessive alcohol intake has been shown to be associated with up to 36% of DCM cases [66]. The postulated pathogenic mechanisms include myocyte hypertrophy and apoptosis, mitochondria damage, oxidative stress injury, intracellular alteration of contractile proteins and calcium homeostasis [67]. Moreover, sympathomimetic drugs (e.g., cocaine, amphetamines, cathinones), chemotherapeutic agents (anthracyclines), and antipsychotic drugs (clozapine, olanzapine) have been shown to be associated with the development of DCM [68].

##### Peripartum Cardiomyopathy

Peripartum cardiomyopathy (PPCM) is a rare, often dilated, cardiomyopathy with heart failure secondary to LV systolic dysfunction affecting childbearing women in late pregnancy or up to 5 months postpartum. Potential risk factors for PPCM include race (mainly Afro-Caribbean descent), multiparity, advanced maternal age and presence of hypertension with or without pre-eclampsia. Although the exact pathogenesis of PPCM is still not fully established, the proposed etiology is multifactorial. A main proposed mechanism suggests an unbalanced peripartum oxidative stress that triggers the cleavage of the nursing hormone prolactin into a potent antiangiogenic and proinflammatory fragment leading to endothelial and cardiomyocyte dysfunction [69]. Recent data suggest the presence of genetic mutation (in particular TTN gene) associated with genetic DCM in around 15–20% of PPCM patients, which supports the crucial role of genetic predisposition in the pathophysiology of PPCM [70].

### 3.2. Clinical Manifestation

Usually, DCM presents typical signs and symptoms of congestive heart failure as progressive dyspnea, ankle swelling and orthopnea. Other forms of initial presentation include arrhythmias (especially in case of LMNA mutations), thromboembolic events and SCD. In fewer cases, asymptomatic cardiomegaly may be the first presenting sign [53].

### 3.3. Diagnosis

#### 3.3.1. Echocardiography

Echocardiography has crucial importance in the diagnosis, follow-up and family screening of DCM. The main echocardiographic diagnostic feature is characterized by presence of LVEF < 45% and LV end-diastolic diameter (LVEDD) or volume greater than two standard deviations according to nomograms corrected by age, gender and body surface area [53]. These findings are usually associated with LV eccentric hypertrophy with normal or mildly increased LV wall thickness, which plays a key role in the differential diagnosis with other structural myocardial abnormalities such as HCM or hypertensive and infiltrative cardiomyopathies.

The presence of right ventricular dilatation and dysfunction correlates usually with a worse functional status and more advanced stages of heart failure [71]. Recent imaging advances in the field of cardiac ultrasound, including analyses of myocardial strain and speckle-tracking deformation represent a promising approach for an earlier disease detection especially in relatives of DCM patients [72].

#### 3.3.2. CMR

CMR offers a highly sensitive assessment of global biventricular size and function. Moreover, CMR enables through LGE assessment detection of myocardial fibrosis and oedema, which allows a differentiation among various causes of DCM [73]. In this context, LV midwall linear fibrosis which may be found in approximately 30% of DCM cases, has been shown to be an independent predictor of all-cause mortality, cardiovascular mortality and development of ventricular arrhythmias [74]. In the light of the increasing role of CMR in risk stratification of SCD, the DERIVATE study as a large multicenter registry evaluated the prognostic value of CMR variables in predicting all-cause mortality and major adverse arrhythmic cardiac events (MAACE) in 1508 patients with non-ischemic DCM with chronic heart failure and a LVEF < 50%. After a mean follow-up period over 2 years, the independent predictors of MACCE were male gender, left ventricular end-diastolic volume index on CMR (LVEDVi), and presence of more than three segments with midwall fibrosis on LGE. On the other hand, only patient age and the presence of mid-wall LGE were independent predictors of all-cause mortality. Based on these results, the authors proposed a risk score which includes as variables male gender, LVEDVi and midwall LGE which in return may allow a reclassification of candidates for primary prevention according to the current conventional criteria for ICD implantation [75].

#### 3.3.3. Coronary Angiography and Endomyocardial Biopsy

Ischemic cardiomyopathy can be differentiated from DCM by echocardiography in the vast majority of cases. However, ischemic cardiomyopathy may present as global hypokinesis mimicking DCM. In these situations, coronary angiography should be considered to exclude ischemic cause, especially in the presence of risk factors for coronary artery disease [76]. In patients with clinically suspected giant cell or eosinophilic myocarditis, EMB including histology, immunohistology and polymerase chain reaction, is recommended [63]. Moreover, endomyocardial biopsy (EMB) should be considered in case of clinical suspicion of systemic, storage or metabolic diseases (e.g., sarcoidosis, hemochromatosis, vasculitis, autoimmune conditions) that may require specific treatments and cannot be excluded in other means [53].

#### 3.3.4. Genetic Counselling and Testing

Currently, the American and European Heart Rhythm Societies recommend genetic testing for DCM patients and their first-degree relatives in the presence of significant conduction disease and/or a family history of SCD. Relatives who do not carry a pathogenic variant are reassured and discharged from ongoing cardiac follow-up. On the other hand, carriers of a familial variant should undergo close clinical surveillance [25,77].

### 3.4. Management Aspects

The management of DCM is focused on reducing congestive heart failure symptoms as well as early surveillance and treatment of arrhythmia.

#### 3.4.1. Pharmacological Therapy

The pharmacological treatment of heart failure in DCM patients with reduced LVEF follows the current guideline-directed therapies which includes angiotensin-converting enzyme (ACE) inhibitors (or Angiotensin receptor blockers (ARBs)), in association with beta-blockers, and mineralocorticoid antagonists [78]. Incremental benefits in terms of reducing mortality and hospital admissions have been shown in patients with heart failure with reduced LVEF treated with angiotensin receptor-neprilysin inhibitor (ARNI) and the sodium-glucose co-transporter 2 (SGLT2) inhibitors [79].

#### 3.4.2. Primary Prevention of SCD

According to the latest 2022 ESC guidelines, optimal heart failure medical therapy is mandatory in all patients with DCM. Re-evaluation of the clinical status after 3 months before primary prevention of SCD by ICD is also required. Accordingly, in patients with symptomatic heart failure with LVEF ≤ 35% due to non-ischemic DCM despite 3 months of optimal medical therapy should be considered for ICD implantation as primary prevention of SCD (Class IIa-recommendation) [24,80]. In a meta-analysis of six primary prevention trials (CAT, AMIOVIRT, DEFINITE, COMPANION, SCD-HeFT, and DANISH trial) in non-ischemic DCM patients with reduced LVEF, ICD therapy was associated with a significant reduction in overall mortality (RR 0.76; 95% CI 0.65–0.91; *p* = 0.002) [81].

The refining of the indication for primary ICD implantation in non-ischemic DCM derived from the challenging results of the DANISH trial which enrolled 1116 patients with symptomatic non-ischemic cardiomyopathy (with LVEF ≤ 35%). The patients were randomized to ICD or no ICD after optimal medical therapy. At a 5-year-follow up, no difference in all-cause mortality between the two groups was observed. Indeed, only one-fifth of patients with SCD had an LVEF ≤ 35%, whereas about 80% of patients with non-ischemic DCM with ICD having an ejection fraction ≤35% did not experience during a 5-year-follow-up any device intervention due to malignant ventricular arrhythmia [82]. Interestingly, one year later a subanalysis of the DANISH study showed that ICD implantation was associated with a linear decrease in all-cause mortality in patients ≤70 years of age (HR, 0.70; 95% CI 0.51–0.96; *p* = 0.03) and no mortality benefit was seen in those older than 70 years. These findings were justified by the fact that patients with advanced age (older than 70 years) are more likely die from non-cardiac comorbidities rather than SCD [83]. Concerning the application of this finding in clinical practice, the authors had explicitly highlighted the fact that 58% of the overall study population had received a CRT-pacemaker device (and in 68% of patients >70 years of age) that in return may have resulted in masking the primary prevention effect of ICD [83].

Regarding carriers of LMNA gene mutations with manifest cardiac phenotype (impaired LVEF, documented atrioventricular conduction disorders, or non-sustained ventricular tachycardia), a class IIa recommendation for primary preventive ICD has been assigned and these patients are discouraged from competitive physical activities [24,80,84].

#### 3.4.3. Approach to End-Stage Heart Failure

Although recent pharmacological advances in the management of DCM have made a major impact on the clinical course and prognosis, DCM patients remain at increased risk of frequent hospitalization and end stage heart failure. In patients with disease course refractory to maximal medical treatment, HTx represents the gold standard therapy in the absence of contraindications. Moreover, implantation of left ventricular assist devices (LVAD) represent a bridge-to-transplant or a destination therapy in those who are not candidates for HTx [85].

### 3.5. Emerging Pharmacological Approaches in Non-Ischemic DCM

The substantial advances in the identification of molecular and genetic markers of DCM highlight the importance of an etiology-oriented approach to therapy. In the context, cell therapy represents a promising treatment modality in non-ischemic DCM (Table 3). Although the majority of randomized clinical trials of cell therapy have been focusing on ischemic heart disease [86], increasing evidence also supports their clinical beneficial effects in non-ischemic cardiomyopathies. The TOPCARE-DCM trial was one of the first studies demonstrating an improvement in LVEF and decrease in NT-proBNP following intracoronary infusion of bone marrow-derived cells (BMCs) in patients with non-ischemic DCM [87]. This study was followed by the ABCD trial including 85 patients receiving either BMCs or medical therapy (control arm). After a mean follow-up of 28 months, there was a significant improvement in LVEF and concomitant decrease in end-systolic volumes [88]. More recently, the randomized, placebo-controlled trial REGENERATE-DCM trial demonstrated an improvement in heart failure symptoms, biochemical parameters (NT-pro BNP), exercise capacity, and quality of life in 60 patients with non-ischemic DCM after administration of granulocyte colony-stimulating factor (G-CSF) with or without adjunctive intracoronary delivery of autologous BMCs [89]. Additional promising findings came from the POSEIDON-DCM trial randomizing 37 patients with non-ischemic DCM to either autologous or allogeneic BMCs derived therapy. Although more prominent in the allogenic therapy group, LVEF improved in both groups [90].

Targeting genetic substrates is another emerging therapeutic approach for myocardial fibrosis, being a major determinant of disease progression and arrhythmia risk in DCM. iPHORECAST is a multicenter, randomized, controlled trial designed to address whether preventive administration of mineralocorticoid receptor antagonist with established antifibrotic effects (eplerenone) can prevent or delay cardiac remodeling and dysfunction in non-symptomatic PLN p.Arg14del carriers. A total of 84 presymptomatic PLN p.Arg14del carriers (*n* = 42 per group) were randomized to receive either 50 mg eplerenone once daily or no treatment. The primary endpoint includes a multiparametric assessment of disease progression based on CMR parameters, electrocardiographic indicators, exercise testing, heart failure symptoms, and cardiovascular deaths over a follow-up period of 3 years [91]. Results are still pending.

As previously stated, accumulating data have revealed that local and systemic inflammation constitutes a pivotal component in the pathogenesis of non-ischemic DCM. A xantine derivative (namely pentoxifylline) that had the potential to actively suppress the tumor necrosis factor alpha (TNFα) secreted by activated macrophages and T-cells was employed in different trials with DCM. Despite promising results in terms of attenuating the apoptotic stimuli and inflammatory markers, these studies have limitations due to the small number of patients and short duration of follow-up [92].

## 4. Arrhythmogenic Cardiomyopathy

### 4.1. Definition

ACM is a genetically determined cardiomyopathy, characterized histopathologically by progressive fibro-fatty infiltration of ventricular myocardium, clinically predisposing individuals to ventricular dysfunction, life-threatening ventricular arrhythmias, and SCD. The pathological process starts from the epicardium and may progress to become transmural, leading to wall thinning and aneurysm formation [93]. In its initial description, the pathognomic features of the disease were described in the inferior wall, apex, and infundibulum of the right ventricle (the so called “triangle of dysplasia”) [94,95]. However, postmortem studies allowed for the identification of biventricular and predominant LV variants [96]. The estimated prevalence of ACM depends on geographical differences and ranges from 1:2000 down to 1:5000 in the general population [96]. Since SCD represents most commonly the initial manifestation, disease prevalence remains underestimated in most populations. Northeastern Italy is historically the region of highest prevalence, where the disease represents a leading cause of SCD cases among young athletes [97]. The disease is more common in males, possibly secondary to hormonal differences, and often has an average age of onset in the second–fourth decades of life [98].

### 4.2. Etiology and Pathophysiology

#### 4.2.1. Genetic Hallmarks

ACM is a genetically heterogeneous disease that follows mainly an autosomal dominant pattern with incomplete penetrance and variable expressivity. In nearly 60% of ACM cases, pathogenic mutations affect genes encoding for components of the cardiac desmosomes. Desmosomes are intercellular adhesive complexes, which together with ion channels and adherent junction components form the composite area responsible for cellular integration, intracellular signaling cascades, and electromechanical coupling between the cardiomyocytes. The most frequently mutant desmosomal proteins include desmoplakin (DSP), plakophilin (PKP2), desmocollin (DSC2), and desmoglein (DSG2) [99]. Such mutations lead to alterations of intracellular signaling pathways and electromechanical connection between cardiomyocytes, favoring myocyte death, inflammation, and fibro-adipogenesis [100]. Non-desmosomal mutations involving genes encoding for cytoskeletal components and ion channels have been reported in ACM leading to disruption of the cytoskeleton and ion channel dysfunction (e.g., desmin (DES), phospholamban (PLN), filamin C (FLNC), transmembrane protein-43 (TMEM43), transforming growth factor (TGF-β3) and cardiac sodium channel (SCN5A) and LMNA gene) (Table 1) [101,102].

Metabolic pathway changes may also be involved in the development of cardiac dysfunction and lipid accumulation of ACM. An impaired fatty acids metabolism was observed in a cardiac-specific Dsg2 knockout mice model. This was at least in part related to the suppression of genes regulating fatty acids uptake and oxidation [103].

#### 4.2.2. Inflammation

In recent years, increasing evidence supports a role of myocardial inflammation in the pathogenesis of ACM. Multifocal inflammatory immune cell infiltrates consisting mainly of lymphocytic interstitial aggregation along with cardiomyocyte death are a common histopathologic finding in ventricular myocardium in human postmortem specimens or EMB of ACM cases [104].

The role of regional myocardial inflammation in ACM patients was also demonstrated noninvasively through the combined analysis of plasma levels of inflammatory cytokines and cardiac scintigraphy [105]. Recently, anti-desmosomal autoantibodies (anti-desmoglein-2) were reported in serum of ACM patients. In the same study, disease severity and risk of arrhythmia were positively correlated with antibody titers [106]. At the molecular level, the presence of activated inflammatory cascade through the upregulation of pro-inflammatory nuclear factor κB (NF-κB) signaling as well as enhancement of release of inflammatory cytokines was observed in cardiac myocytes of ACM murine models [107]. However, it remains unclear whether the inflammatory response could be the main trigger of ACM mutations or is a consequence of cardiomyocyte death due to genetically determined desmosomal disruption.

### 4.3. Clinical Manifestation

A characteristic feature of ACM compared to other forms of cardiomyopathy is the early occurrence of arrhythmia that precedes cardiac dysfunction. ACM encompasses a large spectrum of phenotypic expressions, ranging from asymptomatic disease to symptomatic patients whose first manifestation is ventricular arrhythmias that may lead to SCD. Most commonly, signs and symptoms usually manifest between the ages of 13 and 40 years and include palpitations, syncopal episodes (mainly during physical exercise), and SCD. The natural clinical progression of ACM can be divided in three phases: an initial preclinical phase, which is commonly associated with asymptomatic electrocardiographic abnormalities that can be observed (premature ventricular beats and T-wave inversions) and myocardial structural and functional abnormalities (segmental or global dilatation and dysfunction of the ventricles); an overt phase characterized by symptoms related to ventricular arrhythmias that may progress in SCD; and, lastly, a progressive phase associated with congestion symptoms due to biventricular heart failure [108].

Rarely, ACM may manifest with acute chest pain and increased troponin release, defined as ‘hot phase’, which resembles clinically acute myocardial infarction or myocarditis [104].

### 4.4. Diagnosis

There is no single diagnostic gold standard for ACM. Recently, the 2020 International Expert consensus document provided upgraded criteria (“the Padua Criteria”) emphasizing the diagnostic approach of the entire phenotypic spectrum of ACM. This approach encompasses a scoring system based on the assessment of morphofunctional and structural myocardial abnormalities [109].

#### 4.4.1. Electrocardiogram

Negative T-waves, especially in the anterior precordial leads, are the most common electrocardiographic feature. Repolarization abnormalities may extend to inferolateral leads, especially in the biventricular or left dominant variants. Depolarization changes reflect areas of slow intraventricular conduction, including right bundle branch block, prolongation of QRS-duration, and post-excitation epsilon waves.

The spectrum of ventricular arrhythmias in ACM ranges from frequent ventricular extrasystoles to non-sustained/sustained ventricular tachycardia that may have either left or right bundle branch block morphology [109].

#### 4.4.2. Echocardiography

Echocardiography represents the first-line imaging approach to evaluate the morphofunctional anomalies in patients with suspected ACM. The presence of right ventricular akinesia, dyskinesia, or bulging in association with global right ventricular dilatation and systolic dysfunction represent major criteria in the right ventricular variants of ACM. Furthermore, reduction of LVEF or echocardiographic global longitudinal strain, with or without LV dilatation, may be observed in LV disease variants [109].

#### 4.4.3. CMR

CMR findings with tissue characterization using LGE have been recently introduced as a leading imaging modality mainly for the diagnosis of LV abnormalities in ACM. Structural abnormalities due to fibrofatty myocardial infiltration are characterized by LGE, predominantly affecting the subepicardial inferolateral regions. Interestingly, such regional distribution of LGE may not be necessarily associated with LV motion abnormalities or systolic dysfunction [109].

Furthermore, phenotypic tissue characterization assessed by CMR has an increasing prognostic value in ACM patients. In a recent study including 140 patients with definite diagnosis of ACM, the presence of LV involvement in CMR predicted an increased risk of SCD, appropriate ICD interventions, and aborted cardiac arrest compared to the biventricular or isolated right ventricular forms during a follow-up period of 6 years. Basing on these results, the study suggested an early ICD implantation in ACM patients with dominant LV phenotype on CMR [110].

#### 4.4.4. EMB

EMB is not routinely recommended for diagnosis of ACM and should be reserved for highly select cases where the non-invasive imaging modalities are not conclusive and the final diagnosis depends on histologic exclusion (especially cardiac sarcoidosis) [111].

#### 4.4.5. Genetic Counselling and Testing

Genetic testing is recommended in all cases with suspected ACM as well as in all *first-degree relatives starting from the age of 10 years* to detect disease *at an early preclinical* stage. Moreover, all first-degree relatives of ACM patients should undergo periodical regular clinical evaluation, electrocardiogram, echocardiography, and possibly CMR, every 1–3 years depending on age, activity level, and phenotype [112].

### 4.5. Management Aspects

#### 4.5.1. Lifestyle Changes and Pharmacological Therapy

The main objective in ACM management focuses on the prevention of arrhythmias and SCD, treatment of heart failure symptoms, and slowing of disease progression. Vigorous physical exercise is considered an important risk factor because it accelerates disease progression and triggers life-threatening ventricular arrhythmias. Accordingly, avoidance of high-intensity exercise is recommended in all patients with definite ACM diagnosis [24]. In patients with ventricular arrhythmias, mainly premature ventricular beats and non-sustained ventricular tachycardia, beta-blockers represent the first-line approach. Among antiarrhythmic drugs, sotalol and amiodarone (alone or in combination with beta-blockers) are the most effective drugs with a relatively low proarrhythmic risk.

In the presence of congestive heart failure, the standard heart failure pharmacological intervention, including ACE inhibitors, ARBs, and diuretics, is recommended.

**Figure 2:**
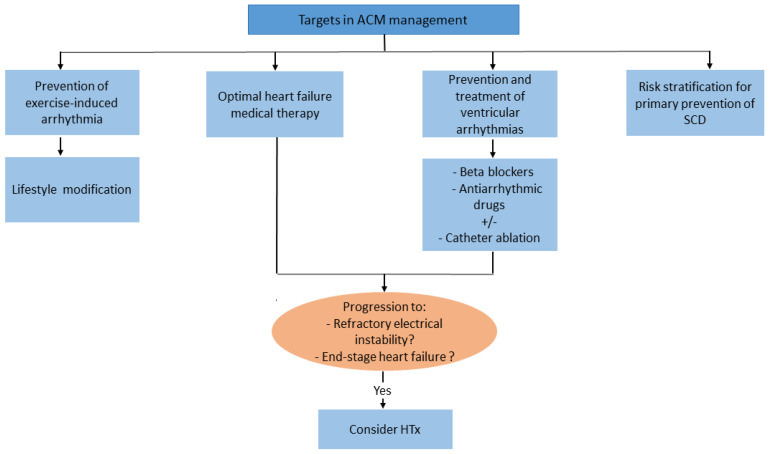
Algorithm summarizing the main treatment objectives for ACM management. ACM: arrhythmogenic cardiomyopathy; SCD: sudden cardiac death; HTx: orthotopic heart transplantation.

HTx represents the final therapeutic option in case of heart failure refractory to be optimal medical therapy or potentially lethal ventricular tachyarrhythmias, which are uncontrollable with antiarrhythmic drugs or catheter ablation-based approaches (Figure 2) [113].

#### 4.5.2. Primary Prevention of SCD

An International Task Force Consensus Statement proposed a risk stratification algorithm for preventive implantation of ICD, based on a review of observational cohort studies in patients with right ventricular variants of ACM [113]. Accordingly, the latest guidelines recommended an ICD implantation (Class I) in all patients with history of ventricular tachycardia/ fibrillation or in presence of severe right or left ventricular dysfunction. Class IIb indication was assigned to patients with ‘minor’ risk factors associated with a risk of major arrhythmic events not sufficiently high to warrant ICD implantation for primary prevention (e.g., male gender, proband status, electrocardiographic changes, amount of LGE, complex genotype, electrocardiographic changes, or inducibility of programmed ventricular stimulation). On the other hand, right ventricular variants of ACM that are asymptomatic or with no risk factors have no indications for prophylactic ICD implantation (Class III-recommendation) [113] (Figure 2).

### 4.6. Promising Pharmacological Therapies in ACM

Current management in ACM patients is limited to symptom relief and prevention of SCD. However, novel therapeutic approaches targeting the underlying genetic and molecular mechanisms are on the horizon.

In preclinical ACM models, application of adeno-associated virus (AAV) gene therapy targeting PLN-gene mutation by delivering antisense ribonucleic acid (RNA) has been shown to restore normal calcium handling [114].

Recent promising findings from a translational study highlighted an involvement of extra-cardiac bone marrow (BM)-mesenchymal stem cells (MSCs) in the pathogenesis of ACM. The examination of both cardiac- and BM- MSCs isolated from DSG2-mutant mice showed profound alterations in cytoskeletal organization, which are directly caused by DSG2 downregulation. Interestingly, the mutant circulating BM-MSCs have shown an increased propensity to migrate to the injured region of the heart [115]. This finding supports the hypothesis that MSC from both cardiac and extra cardiac origin may contribute to myocardial remodeling and fibro-fatty lesion formation in ACM.

## 5. Conclusions and Gaps in Knowledge

In recent years, we have witnessed growing improvements in cardiovascular imaging modalities as well as a deeper knowledge of genotype–phenotype correlations in the field of cardiomyopathies, which in return brought significant advances in the diagnostic and therapeutic decision-making. On the other hand, there are still a number of unanswered questions due to knowledge gaps.

In HCM, SCD remains the most devastating and challenging presentation since it may occur without preceding symptoms. Indeed, the developed risk stratification models adopted by the American and European societies are still imperfect since they are primarily based on registries, retrospective, or prospective small-scale studies.

Another issue is related to the assessment of myocardial fibrosis by CMR through LGE. In fact, there is still no universal standardized technique for the quantification of LGE (most common implied techniques are the six standard deviation method and the full-width at half maximum (FWHM) technique) [116]. Furthermore, understanding the impact of surgical myectomy and alcohol septal reduction on SCD risk is still a challenging point and therefore SCD-prediction model should be cautiously applied in this group. Although cardiac myosin inhibitors are a promising therapeutic option, data supporting the role of these agents in the pediatric HCM population are lacking.

Concerning familial cases of DCM, recommendations for familial cascade screening to identify relatives at risk of developing the disease have been well established by current guidelines. However, there is still no specific evidence for early pharmacological treatment in phenotype-negative carriers of pathogenic genetic variants to prevent LV remodeling or cardiovascular mortality.

Although the fact that around one-third of patients with DCM experience a relapse in chronic disease after an initial recovery of LVEF, current guideline recommendations for DCM treatment still do not vary from general heart failure management [54]. Therefore, it is time to include treatment strategies targeting the molecular causative processes basing on multicenter randomized trials since current evidence in this field is mostly coming from single-center pilot and retrospective cohort studies.

Another remarkable challenge emerged from the refinement of primary ICD indication in non-ischemic DCM by respective guidelines which well delineated the limitation of relying mainly on LVEF [117]. New insights are awaited from the recently started randomized controlled, multi-center CMR-ICD-DZHK23 trial (NCT04558723), randomizing 760 patients with non-ischemic DCM to either optimal medical therapy and implantation of ICD/cardiac resynchronization therapy with a defibrillator (if indicated) versus optimal medical therapy alone. The primary endpoint of the trial is defined as death from any cause during follow-up [118]. Therefore, to further fill knowledge gaps in identifying patients at risk for SCD in non-ischemic DCM, large prospective studies that implement CMR in the early prognostication may be helpful, by also exploiting its capacity to prospectively differentiate responders to therapy from non-responders for medical therapy.

Lastly, a growing body of evidence based on pathological and clinical studies in patients with right-sided phenotypic variants of ACM has provided significant insights into the clinical manifestations, risk stratification, and indications for primary SCD-prevention. However, prospective data on clinical outcome and predictors of arrhythmic burden in patients with biventricular or left-dominant ACM are still lacking in the current guidelines’ recommendations. Another issue to overcome concerns the effectiveness and safety of applying catheter ablation techniques in the management of atrial arrhythmias in ACM. Further, psychosocial stress has been recently suggested as an independent risk factor involved in the pathogenesis of ACM by promoting disease penetration [119]. In the light of these findings, further studies are warranted to elucidate how psychosocial stress as well as cognitive capacities and mood control may mediate the structural and functional deterioration in ACM.

## Figures and Tables

**Table 1:** Summary of the main causative genetic mutations involved in the pathogenesis of HCM, DCM, and ACM.

Gene	Protein	Cardiac Phenotype
MYBPC	Myosin-binding protein C	HCM
MYH7	Myosin heavy chain 7	HCM, DCM
TNNI3, TNNT2	Cardiac troponin I and T	HCM, DCM
LMNA	Lamin A/C	DCM, ACM
PLN	Phospolamban	DCM, ACM
FLNC	Filamin C	DCM, ACM
DES	Desmin	DCM; ACM
ACTC	Actin alpha cardiac muscle 1	DCM
SGCD	Delta-sarcoglycan	DCM
DSP	Desmoplakin	ACM
PKP2	Plakophilin-2	ACM
DSC2	Desmocollin-2	ACM
DSG2	Desmoglein-2	ACM
TMEM43	Transmembrane protein 43	ACM
TGF-β3	Transforming growth factor-β3	ACM
SCN5A	Cardiac sodium channel	ACM

**Table 2:** Highlights related to trials on novel therapeutic approaches for HCM.

Trial	Phase	Agent	No. of Patients	Patient Category	Key Findings
PIONEER-HCM (prospective, open label)	2	Mavacamten	20	Obstructive HCM with resting LVOT gradient ≥ 30 mmHg	↓ Exercise-induced LVOT gradient ↓ Dyspnea scores ↑ Peak oxygen consumption
Explorer-HCM (prospective, randomized, double blind)	3	Mavacamten	251	Obstructive HCM with NYHA class II-III	↓ Exercise-induced LVOT gradient ↑ Peak oxygen consumption ↓ NYHA class
VALOR-HCM (prospective, randomized, double blind)	3	Mavacamten	112	Obstructive HCM referred for septal reduction procedure	↓ Exercise-induced LVOT gradient ↓ NYHA class ↓ need septal reduction therapy
MAVERICK-HCM (prospective, randomized, double blind)	2	Mavacamten	59	Nonobstructive HCM with NYHA class II-III	No significant change in Peak oxygen consumption or NYHA class ↓ NT-proBNP ↓ High-sensitivity troponin
SEQUOIA-HCM (prospective, randomized, double blind)	3	Aficamten	270 (estimated)	Symptomatic obstructive HCM	Ongoing study, aims to: Evaluate changes in peak oxygen consumption, NYHA class, post-valsalva LVOT gradient
REDWOOD-HCM (prospective, randomized, double blind)	2	Aficamten	41	Obstructive HCM with NYHA class II-III	↓ Resting and provocable LVOT gradient (dose-dependent) ↓ NYHA class
RESTYLE-HCM (prospective, randomized, double blind)	2	Ranolazine	80	Nonobstructive HCM	No significant improvement in peak oxygen consumption, exercise tolerance or quality of life ↓ arrhythmic profile
LIBERTY-HCM (prospective, randomized, double blind)	2	Eleclazine	172	Obstructive HCM NYHA class I-IV	No significant improvement in exercise tolerance

Abbreviations: HCM: hypertrophic cardiomyopathy; NYHA: New York Heart Association; LVOT: left ventricular outflow tract; NYHA: New York Heart Association; NT-proBNP: N-terminal pro-brain natriuretic peptide; ↑ increase; ↓ decrease.

**Table 3:** Summary of relevant trials related to pharmacologic approaches in non-ischemic DCM.

Trial	Phase	Agent	No. of Patients	Follow-Up (Months)	Patient Category	Key Findings
iPHORECAST (prospective, randomized, open-label)	III	Eplerenone	60	36	Phospholamban R14del Carriers with NYHA II and LVEF ≥ 40%	Results pending, aims to: assess CMR parameters (functional and morphological), changes in electrocardiographic indicators and NYHA class, cardiovascular death
TOPCARE-DCM (prospective, open-label)	II	Intracoronary infusion of BMCs	33 (33 treated, no control)	12	NYHA I-III and LVEF ≤ 40%	↑ LVEF
ABCD trial (prospective, randomized, open-label)	II	Intracoronary infusion of BMCs	85 (45 treated, 40 control)	6	NYHA ≥ II and LVEF ≤ 40%	↑ LVEF ↑ Quality of life ↔ Mortality
REGENERATE-DCM (prospective, randomized, placebo-controlled, double-blind)	II	Intracoronary infusion of BMCs and peripheral G-CSF	60 (30 treated, 30 control)	12	NYHA ≥ II and LVEF ≤ 45%	↑ LVEF ↓ NYHA class ↑ exercise capacity
POSEIDON-DCM (prospective, randomized, open-label)	I/II	autologous or allogeneic BMCs	37 (19 allo BMC, 18 auto BMC)	12	LVEF < 40% and LVEDD > 5.9 cm in males and > 5.6 cm in females	↑ LVEF

Abbreviations: BMCs: bone marrow-derived cells; CMR: cardiac magnetic resonance; DCM dilated cardiomyopathy; G-CSF: granulocyte colony-stimulating factor; LVEF: left ventricular ejection of fraction; NYHA: New York Heart Association; ↑: increase; ↓: decrease; ↔: no change.

## Data Availability

Not applicable.

## Abbreviations

AAV	adeno-associated virus
ACTC1	actin-alpha cardiac muscle
ACE	angiotensin-converting enzyme
ACM	arrhythmogenic cardiomyopathy
ACTC	cardiac actin
ATPase	adenosine triphosphatase
ARBs	angiotensin receptor blockers
ARNI	angiotensin receptor-neprilysin inhibitor
ACC	American College of Cardiology
AHA	American Heart Association
BMCs	marrow-derived cells
BM-MSCs	bone marrow mesenchymal stem cells
CAD	coronary artery disease
CMR	cardiac magnetic resonance
COVID-19	coronavirus disease 2019
DCM	dilated cardiomyopathy
DES	desmin
DMD	dystrophin
DSC2	desmocollin
DSG2	desmoglein
DSP	desmoplakin
ESC	European Society of Cardiology
EDMD	muscular dystrophy Emery-Dreyfus
EMB	endomyocardial biopsy
ECV	extracellular volume
FDA	U.S. Food and Drug administration
FWHM	full-width at half maximum
FLNC	filamin C
G-CSF	granulocyte colony-stimulating factor
HbA1c	haemoglobin A1c
HCM	hypertrophic cardiomyopathy
HTx	orthotopic heart transplantation
IL-1β	Interleukine-1β
ICD	implantable cardioverter-defibrillator
LVEF	left ventricular ejection of fraction
LVEDVi	left ventricular end-diastolic volume index
LVEDD	left ventricular end-diastolic diameter
LGE	late gadolinium enhancement
LMNA	Lamin A/C
LRP6	low density lipoprotein receptor-related protein 6
LVAD	left ventricular assist devices
LVOT	left ventricular outflow tract
MAACE	mortality and major adverse arrhythmic cardiac events
MYH7	beta-myosin heavy chain
NT-proBNP	N-terminal pro-brain natriuretic peptide
NF-κB	nuclear factor-κB
NYHA	New York Heart Association
PKP2	plakophilin
PLN	phospholamban
PPCM	peripartum cardiomyopathy
SAM	systolic anterior motion of the mitral valve
SCD	sudden cardiac death
SGCD	delta-sarcoglycan
SGLT2	sodium-glucose co-transporter 2
TDI	tissue Doppler imaging
TNNI3	troponin I
TNNT2	troponin T
TTN	titin
TDI	tissue Doppler imaging
TNFα	tumor necrosis factor alpha
↑	increase
↓	decrease
↔	no change
